# Exploring Factors Associated With the Motivation of Clinical Pharmacists: A Focus on the South African Context

**DOI:** 10.3389/fmed.2021.747348

**Published:** 2021-11-23

**Authors:** Lucille Crafford, Anouk Wouters, Elmien Bronkhorst, Andries G. S. Gous, Rashmi A. Kusurkar

**Affiliations:** ^1^Division of Clinical Pharmacy, School of Pharmacy, Sefako Makgatho Health Sciences University, Pretoria, South Africa; ^2^Amsterdam University Medical Centers, Research in Education, Faculty of Medicine Vrije Universiteit Amsterdam, Amsterdam, Netherlands; ^3^LEARN! Research Institute for Learning and Education, Faculty of Psychology and Education, Vrije Universiteit Amsterdam, Amsterdam, Netherlands

**Keywords:** clinical pharmacy, motivation, Self-determination Theory (SDT), Academic Motivation Scale (AMS), pharmacist

## Abstract

**Introduction:** Pharmacy practice in many middle to low-income countries has slowly transitioned from being product-focused to a more patient-focused clinical practice. Lack of motivation is one of the factors contributing to the scarcity of pharmacists in the wards. As little is known about motivation in clinical pharmacists, this study aimed to obtain insight into the quantity and quality of their work motivation and factors associated with it.

**Methods:** Self-determination Theory, used as the framework, describes autonomous motivation as being generated from within or through personal endorsement and controlled motivation as originating from external factors. An online questionnaire including the Academic Motivation Scale to measure autonomous motivation, controlled motivation and amotivation, was sent to clinical pharmacy graduates from 2000 to 2020 across South Africa, followed by interviews to explain some results. Independent *t*-test was used to analyze differences in motivation of clinical pharmacists to perform clinical services based on personal and environmental factors. Interview data were transcribed and analyzed to explain significant quantitative findings.

**Results:** Higher amotivation was found in graduates who are currently not practicing in dedicated clinical pharmacist positions, as well as in graduates who do not receive additional financial benefits for clinical services. We found no significant differences in the work motivation of clinical pharmacists based on their gender, age, current practice setting, work experience and additional training received. The interviews revealed that relatedness and autonomy are the most important factors for clinical pharmacists' work motivation.

**Discussion:** Overall participants had a high mean autonomous motivation, a high mean controlled motivation and low mean amotivation. In line with Self-determination Theory literature, considering the basic psychological needs for relatedness and autonomy could assist with designing interventions, like creating a supportive work environment, to optimize motivation. This could improve professional wellbeing, service implementation and prevent possible adverse events. Future research is necessary to understand barriers and facilitators of clinical pharmacists' work motivation.

## Introduction

The implementation of pharmaceutical care through patient-centered clinical pharmacy services improves the rational use of medications resulting in better patient outcomes (1–4). Despite the clear benefits, the transition from product-focused to patient-focused pharmacy practice has been rather slow in many middle to low-income countries. This slow uptake of clinical pharmacy services in such countries is generally related to infrastructure and resource limitations [(5–7)]. Additionally, lack of motivation has been identified in South Africa (SA) as a factor for the scarce number of pharmacists working in the wards (8). In spite of this, there is a lack of qualitative research on the work motivation of medical specialists (9), especially investigation into the motivation of clinical pharmacists. Limited data are available on the application of motivational theories to address motivation-related issues among healthcare workers in Africa. In this study we aimed to explore the motivation of clinical pharmacists, and differences in motivation in relation to personal and professional factors/characteristics, in order to provide the opportunity to create the best possible work environment for them.

**Figure 1:**
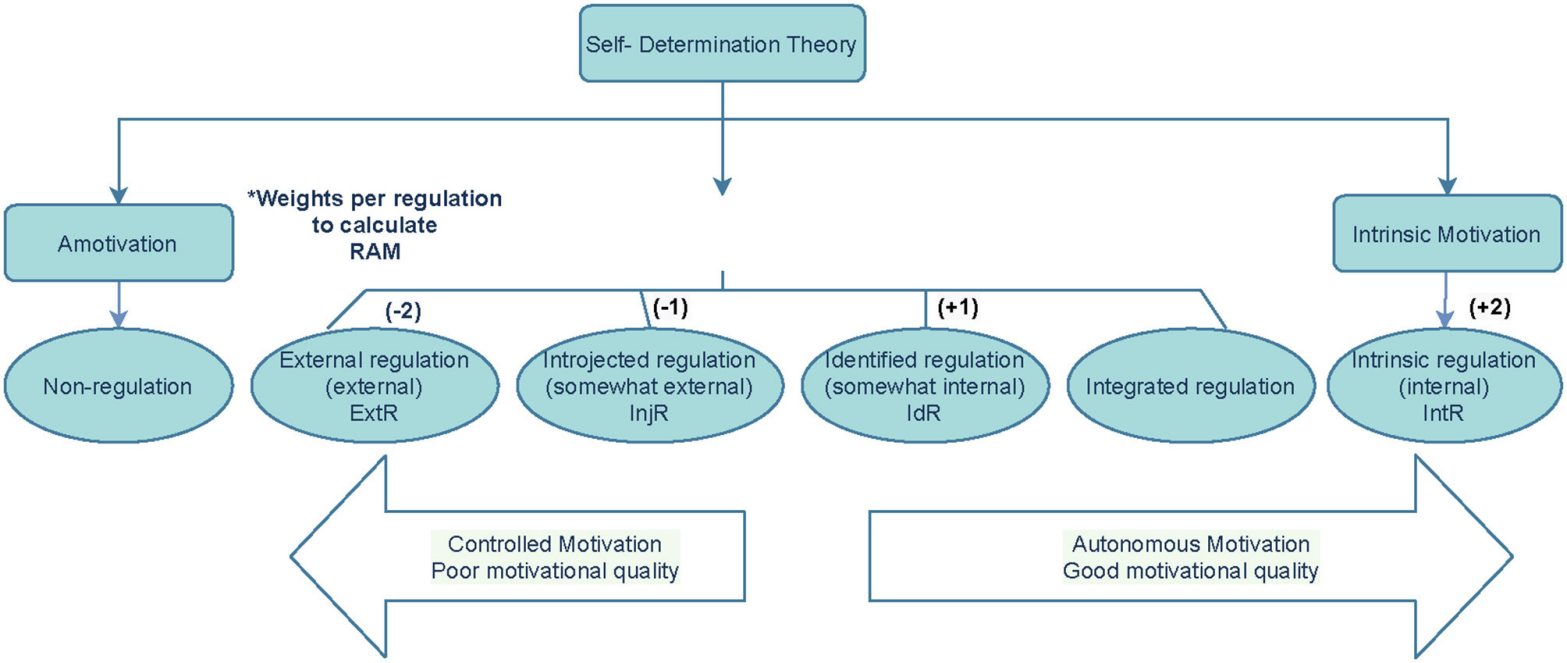
Illustration of the Self-determination Theory adapted from Deci and Ryan (12). *Relative Autonomous Motivation (RAM) = (−2ExtR) + (−1lnjR) + (1xldR) + (2xlntR).

The motivation of professionals plays a vital role in professional development and in their overall quality of work and performance (9, 10). Self-determination Theory (SDT) is among the current major motivational theories in psychology, as well as in Health Professions Education. Research on SDT has been applied effectively to examine what motivates individuals to work, however there is a scarcity of existing literature on work performance motivation and its determinants (9), as well as the use of SDT in the profession of clinical pharmacy. SDT describes motivation as dynamic and dependent on three basic psychological needs (BPNs), i.e., autonomy (experiencing a sense of volition), competence (feeling capable of achieving targeted outcomes) and relatedness (feeling connected to others). This theory describes motivation along a continuum that ranges from amotivation to intrinsic motivation (IM) (11) as illustrated in Figure 1.

The SDT does not solely quantify motivation, but also emphasizes the importance of the quality of motivation, classifying two types of motivation; autonomous motivation (which includes identified regulation and intrinsic motivation and is described as being generated from within or through personal endorsement), and controlled motivation (which includes external and introjected extrinsic motivation (EM) and is described as being generated from external or internal pressure or rewards) (12). Autonomous motivation (AM) has been associated with better performance, more positive well-being and greater resilience among health care professionals (13, 14). Controlled motivation (CM) is not the desired motivation type and is associated with burnout (10, 15). Amotivation, however, is the complete absence of motivation or purpose (12).

Factors previously found to influence the motivation of health care professionals are patient care, working with colleagues, work environment, technical issues, organizational and administrative tasks, job content and financial benefits (9, 16). Similar to any other healthcare profession, the universal performance of clinical pharmacists depends on their motivation and job satisfaction derived from certain intrinsic and extrinsic motivators (17–19). Where motivation is negatively influenced, feelings of emotional frustration are evoked, in turn decreasing the well-being, performance and, thus the quality of patient care (9). Additionally, job dissatisfaction is related to burnout, which is associated with negative effects on professional well-being (19–22).

The profession of pharmacy is maturing as a clinical profession in South Africa (SA) and has experienced significant development and growth over the past 10 years (8). However, as of yet, the discipline cannot be registered as a specialization at the South African Pharmacy Council (SAPC), and as a result no official clinical pharmacist posts are available (8). The healthcare system in SA constitutes a two-tiered health system, that is the public and private health care systems, serving ~84 and 16% of the country's population, respectively (23). South Africa, additionally, experiences human resource challenges in terms of healthcare service delivery. The shortage of pharmacists, especially in the hospital environment, has been acknowledged (24–26), leading to an increased burden on institutional pharmacists to provide product-focused tasks, like dispensing (24). Clinical pharmacists have not yet been appointed in allocated posts in the public sector, and the private sector has created ward-based pharmacist or clinical practice pharmacist posts, to bridge the gap. However, each hospital group or institution has its own post/job descriptions, also with differences in time available to spend in the wards for the delivery of pharmaceutical care. This can be explained by the existing human resource shortage, combined with the suboptimal use or lack of technical support staff (24, 26) which makes it challenging to allocate pharmacists to work in a clinical ward on a daily basis. Moreover, not being fully integrated in the hospital environment, clinical pharmacists cannot optimally fulfill their role in providing adequate pharmaceutical care (27). The COVID-19 pandemic has further complicated this; movement beyond the hospital pharmacy has been limited because pharmacists have been preferably allocated to deliver dispensing-like services. As clinical pharmacy in SA still faces numerous challenges regarding its implementation, the shortage of pharmacists with the necessary clinical expertise in turn leads to an increased need for clinical pharmacy services. The involvement of clinical pharmacists in providing services like medication reconciliation, antibiotic stewardship and anticoagulant monitoring highlights their role in the multidisciplinary team and shows how clinical integration impacts the provision of pharmaceutical care (2, 28–31).

In a study by Bronkhorst et al. (8), pharmacists across SA experienced some form of barrier to perform their role optimally, with the main barrier perceived to be lack of dedicated time to deliver clinical services. Furthermore, this study suggested that additional investigation into the work motivation for clinical pharmacists, may be necessary to establish the success of a practice Two other studies also found that clinical pharmacists are not involved in ward rounds, because they are over-burdened with other work responsibilities (32, 33). Nevertheless, globally it was recorded that within a fully implemented model of integrated pharmaceutical care, the change in scope relieves the clinical pharmacists of their dispensing responsibility, enabling them to work fulltime delivering clinical pharmacy services (2, 31, 34, 35).

The purpose of the present study was to investigate the motivation of clinical pharmacy graduates to perform clinical pharmacy-related services, and what personal (demographics), and work environment factors are associated with their motivation. Accordingly, the research questions for this study were as follows: (1) How can the quality of motivation (AM/CM) of clinical pharmacists to perform clinical ward-based activities be characterized? (2) Which factors are related to clinical pharmacists' quality of motivation (AM/CM)?

Our study contributes to the literature by validating SDT in a target group that has not been studied before, and where motivation could be a key factor in establishing the success of clinical pharmacy practice in middle to low-income countries.

## Methods

### Study Design

This study followed a mixed methods approach with an explanatory design (QUANT-qual). In 2019, an electronic self-report questionnaire was administered *via* Typeform™ to all clinical pharmacy graduates from the year 2000 to 2018. Two years later, in 2021, the electronic self-report questionnaire was again administered, to include the most recent graduates of 2019 and 2020, sequentially increasing the original sample size. Follow-up semi-structured qualitative interviews were conducted to explain some of the unexpected quantitative findings. In this study, we defined a “clinical pharmacist” as one who has completed the Master of Pharmacy (M. Pharm) in Clinical Pharmacy degree.

### Educational Context

In SA, the entry-level pharmacy degree consists of a 4-year Bachelor of Pharmacy (B. Pharm) qualification. Graduates are thereafter required to complete an internship year under supervision of a registered pharmacist prior to their own registration as a pharmacist. The SAPC currently approves nine pharmacy schools to train pharmacists, and recognizes two specialties: radiopharmacist and clinical pharmacokineticists (7). While registration of clinical pharmacy as a specialty is still pending, specialist registration is nonetheless an identified priority for development (36). In anticipation, the National Department of Health have already created clinical pharmacy specialization posts (37, 38), and a number of M. Pharm programmes are offered by universities in SA.

The Sefako Makgatho Health Sciences University (SMU), previously University of Limpopo (MEDUNSA campus), together with the University of Witwatersrand are the only universities that offer the M. Pharm in Clinical Pharmacy degree which includes a mini-dissertation, as well as a modular component. The highest number of students graduate from SMU annually, with an average of 5–6 graduates from 2000 to 2020.

### Practice Context

Pharmacists who have completed the clinical pharmacy qualification, as well as general pharmacists (often enrolled for the part-time M. Pharm degree), perform pharmaceutical care to some degree in the wards. In SA, no standardized clinical activities are set out in different healthcare facilities/hospital settings, and activities mainly include antimicrobial stewardship, promoting the appropriate use of antimicrobials. Other clinical activities performed by pharmacists at ward level include therapeutic drug monitoring, taking ward rounds with the multidisciplinary team, doing medication reconciliation and providing education to patients and nursing staff. A great difference has been reported in the standard and quality of these activities between different settings/facilities (8).

### Study Population and Sample Recruitment

Data for this research were collected from graduates of the M. Pharm degree in the Clinical Pharmacy offered at SMU (7, 39). Contact details of all 114 graduates (2000–2020) were retrieved from the SMU School of Pharmacy database. No restrictions were applied based on whether or not they are currently practicing as clinical pharmacists, in either the public or private healthcare sector.

### Data Collection Instrument

The 28-item Academic Motivation Scale (AMS) (40) was used to measure motivation. This includes measuring the seven subscales, namely IM- to know; IM- toward accomplishment; IM- to experience stimulation; EM- identified; EM- introjected; EM- external regulation and amotivation. The measurement has shown internal consistency, as it allows a valid and reliable assessment of motivation as described by SDT (40, 41) with Cronbach alpha's reported ranging from 0.77 to 0.90 (42). We determined that through adaptation of the scale to the clinical pharmacists' context it was the most suitable instrument to measure the quantity and quality of clinical pharmacy graduates' motivation for delivering clinical pharmacy services, with items like; e.g., “For the pleasure that I experience when delivering clinical pharmacy related activities.” Responses were made on a 5-point Likert scale. AM was calculated by averaging the scores of intrinsic motivation and identified regulation. CM was calculated by averaging the scores of introjected regulation and external regulation (12, 43, 44). Furthermore, the scoring system used in the AMS to determine an individual's motivation is known as Relative Autonomous Motivation (RAM), the measurement of AM relative to CM. The RAM was calculated by assigning different weights to the different types of motivation and summing these scores (see Figure 1).

The questionnaire furthermore collected data on demographic information, previously shown to be related to motivation like age, gender and work experience (45–47). Data on the graduates' current work environment, whether they received additional training and additional incentives for delivering clinical pharmacy services, as well as their duration of practice was also collected. Additional questions explored whether their current work setting has motivational methods in place and which of these methods the participant considers to be effective. The questionnaire was piloted among M. Pharm students and pharmacists to gain insight into how the items on the questionnaire was interpreted. The distribution of responses were examined, and adaptation of the questionnaire conformed to published guidelines (48).

The videoconferencing application Zoom was used for the qualitative *post-hoc* interviews. As the questionnaire was answered anonymously six participants were randomly selected from both the public and private health care sector. The interviews included two questions to explain some significant findings from the questionnaire. A third follow-up question was to understand how participants perceived some of the motivational method options listed. This was done in order to cluster the motivational methods explored in the quantitative questionnaire into methods that are expected to either stimulate AM or CM. It was necessary to establish whether those specific options function as extrinsic or intrinsic motivators.

### Ethical Approval

Ethical approval for this study was received by the Sefako Makgatho Health Sciences University Research and Ethics Committee (SMUREC/P/232/2020:PG). Participation in the survey was voluntary, no incentives were given for participating and responses were anonymized. All participants gave their informed consent for participation in the study.

### Analysis

Descriptive statistics such as the means, frequencies and percentages of the variables were calculated, to assess demographic data such as gender and years of experience. A Cronbach alpha was determined for all subscales to investigate the reliability and internal consistency. Data were checked for normality distribution. These analyses were performed using the Statistical Package of Social Sciences (SPSS) version 26.0. A confirmatory factor analysis (CFA) was done for the seven subscales of the AMS, determining the validity of the questionnaire used. Further analysis testing for variances between independent demographic variables with regards to the dependent motivational dimensions was done using standard deviations obtained from the independent *t*-test of variances. Audio-recordings of the interviews were transcribed and thematic analysis was performed by two of the researchers to clarify how some of the survey questions were perceived and further explain other significant quantitative findings.

## Results

Out of 114 graduates, 74 completed the survey (response rate 65%). This includes 60 participants (from the 97 graduates of 2000–2018) part of the first data collection wave in 2019; and 14 participants (from the 17 graduates of 2019 and 2020) part of the data collection in 2021.

The standardized Cronbach alphas were 0.91, 0.84, and 0.82 for AM, CM, and amotivation, respectively, indicating a high level of internal consistency. The assumption of a normal multivariate distribution was met. A CFA was conducted on the AMS as this was not validated among clinical pharmacists before. Factor loadings above 0.4 are considered good or significant, all factor loadings were adequate, ranging between 0.418 and 0.927, and are presented in Table A1. These results, [together with the Root Mean Square Error of Approximation (RMSEA) of 0.038] suggested that the AMS constituted a valid and useful scale to measure motivation according to the multidimensional perspective of SDT.

Table 1 shows the mean overall motivation of the respondents, as well as the population's demographics with their corresponding mean scores on types of motivation. Differences between mean scores were tested for significance by using a *t*-test.

**Table 1:** Mean scores of clinical pharmacy graduates on autonomous motivation (AM), controlled motivation (CM), and amotivation.

**Characteristic**	**No. respondents, *n* (%)**	**Mean amotivation (SD)**	**Mean CM (SD)**	**Mean AM (SD)**	**Mean RAM (SD)**
Total sample	74	1.57	3.07	4.22	3.81
**Gender (*****n*** **=** **74)**					
Female	54 (73%)	1.57 (0.776)	2.99 (0.799)	4.17 (0.569)	3.86 (2.873)
Male	20 (27%)	1.59 (0.875)	3.30 (0.901)	4.37 (0.574)	3.67 (2.422)
**Age (*****n*** **=** **60)**					
≤29 years	15 (25%)	1.42 (0.540)	3.16 (0.911)	4.30 (0.582)	3.75 (3.580)
>29 years	45 (75%)	1.54 (0.812)	3.07 (0.867)	4.23 (0.600)	3.87 (2.542)
**Current practice setting (*****n*** **=** **74)**					
Public Institution	40 (54%)	1.59 (0.894)	3.10 (0.929)	4.27 (0.617)	3.82 (3.101)
Private Institution	34 (46%)	1.56 (0.680)	3.05 (0.715)	4.17 (0.520)	3.80 (2.298)
**Currently practicing in a clinical pharmacist position (*****n*** **=** **74)**					
Yes	23 (31%)	1.12 (0.475)	3.14 (0.742)	4.36 (0.464)	4.20 (2.263)
No	51 (69%)	1.74 (0.860)	3.05 (0.875)	4.16 (0.610)	3.64 (2.938)
		^*^P = 0.005			
**Work experience (*****n*** **=** **74)**					
<5 years	62 (84%)	1.56 (0.742)	3.06 (0.854)	4.23 (0.599)	3.89 (2.714)
≥5 years	12 (16%)	1.65 (1.079)	3.17 (0.737)	4.22 (0.437)	3.43 (2.982)
**Received additional clinical pharmacy-related training in the work setting (*****n*** **=** **74)**					
Yes	34 (46%)	1.51 (0.624)	2.99 (0.743)	4.23 (0.537)	4.15 (2.528)
No	40 (54.1)	1.63 (0.925)	3.15 (0.904)	4.22 (0.609)	3.53 (2.916)
**Receiving additional income benefits (*****n*** **=** **74)**					
Yes	8 (11%)	1.09 (0.265)	2.91 (0.537)	4.24 (0.469)	4.57 (2.089)
No	66 (89%)	1.63 (0.822)	3.10 (0.862)	4.22 (0.588)	3.72 (2.811)
		^*^P = 0.013			
**Motivational methods in place (*****n*** **=** **73)**					
Motivational methods that stimulate AM	51 (69%)	1.52 (0.777)	3.12 (0.765)	4.24 (0.548)	3.72 (2.405)
Motivational methods that stimulate CM	23 (31%)	1.68 (0.850)	2.97 (0.975)	4.19 (0.638)	4.01 (3.429)

*Mean scores are based on the AMS with a 5-point Likert scale, on which 1 represented strongly disagree and 5 represented strongly agree*.

* *p < 0.05*.

The study comprised of a relatively young population, only 12.2% respondents were ≥40 years.

Higher amotivation was found among graduates not practicing as clinical pharmacists compared to those employed in a dedicated position (*p* = 0.005). Higher amotivation was also found for graduates who do not receive additional financial benefits for delivering clinical pharmacist-related services (*p* = 0.013). No other significant relationships were found, motivation was not associated with age, gender, work setting, years of work experience, whether additional clinical pharmacy related training was received, nor whether motivational methods are in place in the work setting.

The motivational methods, clustered into those that either stimulate AM or CM are shown in Table 2.

**Table 2:** Motivational methods perceived to be the most effective.

**Motivation methods**	***n* = 74 (%)**
**Motivational methods that stimulate AM**	
•Job satisfaction (satisfaction of work related needs)	15 (20%)
•Work environment (co-operative relationship with colleagues, experience feeling of safety)	9 (12%)
•Setting work-related goals to achieve specific outcomes	11 (15%)
**Motivational methods that stimulate CM**	
•Incentives/rewards (i.e., if work performance is excellent)	33 (45%)
•Recognized achievements (i.e., employee of the month)	6 (8%)

Overall, the motivational methods that stimulate CM were perceived to be the most effective (53%), especially incentives given for excellent work performance.

Qualitative data was collected *post-hoc* to elucidate some of the results. Participants answered questions on how their employment status influences their work motivation (whether employed as a dedicated clinical pharmacist or not). They also answered questions on the influence of receiving an additional income for delivering clinical pharmacy-related services on their work motivation. How they perceived the motivational method options “Incentives/rewards” and “Recognized achievements,” provided as part of the survey questions, was also explored.

Detailed findings from the analysis, with quotes from the interview data are presented in Table 3 below.

**Table 3:** Interview results [including quotes of the participants (*n* = 6) of the semi-structured interviews].

**(1) Influence of a dedicated clinical pharmacist position on motivation to deliver clinical pharmacy-related activities**
Support and perceived scope of practice of a clinical pharmacist.
All participants, whether employed in a dedicated clinical pharmacist position or not, indicated that their position influences their motivation. Most graduates acknowledged the lack of support from pharmacy colleagues, other health care professionals, and management, when not employed in a dedicated position as a challenge and having a negative influence on their motivation to practice.
“We have very structured clinical program…there's a great deal of support from my line manager, the pharmacy manager, the hospital manager, the dispensary team, and also regional. So when you have that kind of support, then it makes a little bit easier to perform your job.”
“If you work for a company that supports clinical pharmacy and when you're actually employed in that position, it will help, but what will also help motivate more is to actually do what you studied to do as a clinical pharmacist, compared to being a clinical pharmacist, but only doing antimicrobial stewardship—that's very demotivating. So it depends on where you're working and also the support you get from the work.”
“Not being in a clinical pharmacist position is actually very limiting on the activities that we can actually do… because there's challenges around our other pharmacy colleagues, they don't really understand…even today, there isn't any support and they will say that we are not in a clinical pharmacist position. And if you look at the structure of the pharmacy, it is done in such a way that the capacity has been measured on pharmacist position. So if we move out of the pharmacy to do those clinical activities, they believe that the work that we leave behind will probably suffer, but which isn't the case.”
“I think that the people at hospital management level understand what a clinical pharmacist should do, and try to support, but the pressure you get from head office and what they expect of you is different. Their idea of what a clinical pharmacist does is different to what we perceive to be actually important. Another influence is the input from the doctors and the nurses, because when they understand what you do, it helps to motivate you a bit more than them not knowing what you're supposed to do.”
**(2) Influence of additional income for delivering clinical pharmacy-related services on motivation to practice**
Receiving an additional income for practicing as a clinical pharmacist influences the motivation of graduates to practice.
Factors mentioned that determine whether an additional income is received and the amount thereof included the specific post description, the size of the hospital (hospital beds) and whether goals in the structured joint performance management (JPM) were reached throughout the year.
“You'd get paid as a dispensing pharmacist…and then there's a different salary, for a clinical pharmacist, but which is on the same level as a senior pharmacist.”
“We also have a structured joint performance management, and when you reach all those goals throughout the year, then you get a better increase.”
“You then get a bigger increase than other people, because you managed to reach all your goals in the year.”
However, receiving an additional income did not necessarily lead to higher/better quality motivation.
“Currently, like we are doing, we have to cover retail and the pharmacy floor, and the clinical side. So that little bit more money you're getting doesn't really make up for everything you actually do and doesn't increase my motivation.”
**(3) Motivational methods in place in current work setting**
Clarifying how the following options specified were perceived: (a) “Incentives/ Rewards”, and (b) “Recognized achievements”?
The option “incentives/rewards” according to the six interviewees are associated with money, whether in the form of a monetary reward, or days' leave.
Examples of recognized achievements were verbal recognition of achievements or a rewards programme.
“I perceive it as financial incentive, and recognized achievements would be an email or verbal recognition for excellent performance.”
“Recognized achievements would be any sort of acknowledgment that comes from management level, whether it's verbal or an email or at some meeting or forum where they verbally give you recognition. Then we also have a rewards and recognition programme, where people can nominate you for the work that you've done and they have to write a little motivation as to why they feel you should get that sort of recognition.”

All of the interviewees confirmed that the position they are employed in has an influence on their motivation. Those who were employed in a clinical pharmacist position explained that being employed in a full time capacity enables them to deliver clinical services and knowing what is expected of them, motivates them to practice. This shows how autonomy needs are to some extent fulfilled in dedicated positions. In turn, the interviewees employed in dispensing pharmacist positions explained that they would have been more motivated to do clinical activities if they were in dedicated posts, highlighting some of the challenges like lack of support from colleagues and management, as well as incomprehension of their role by doctors and nurses. This lack of support explains how the quality of motivation for practice depends on an environment that fosters a feeling of relatedness.

While not all graduates received additional income to deliver clinical services, most graduates confirmed that a monetary incentive influenced their motivation. However, one graduate admitted that the extra financial benefit is not enough to increase motivation for practice.

The motivational method “incentives/rewards,” as included in the survey, was associated by all interviewees with a monetary incentive or reward (like a salary increase or extra leave). Furthermore, the participants associated “recognized achievements” with, for example, “a certificate to recognize work done well,” or through acknowledgment from management such as “an email going out to the hospital group saying these people have done this.” The motivational method “recognized achievements” was also identified to stimulate controlled motivation, as it was associated to function as an extrinsic motivator in the form of a reward, as well as an extrinsic motivator in stimulating a feeling of being visible to others.

## Discussion

To our knowledge, this is the first study reporting the quality and quantity of clinical pharmacists' motivation for delivering clinical services in middle to low-income countries.

This study found high overall motivation among clinical pharmacists in SA. This includes a higher mean AM score, as well as higher mean CM score, compared to a study in the Netherlands (47), which measured the quality of motivation of pharmacists for continuous education. This finding raises a concern, because according to SDT, high AM combined with a high CM is associated with burnout (49). Sequentially, burnout, again leads to possible professional consequences like higher chances of quitting the field of work (50), and an overall negative effect on professional well-being and advancement of the clinical pharmacy profession (19–22). Participants furthermore presented with a low mean score on amotivation, corresponding with the low amotivation found in pharmacists in the Netherlands (47). General SDT literature suggests that supporting or thwarting one or more of the three psychological needs, in turn influences the degree to which motivation is autonomous, and the concomitant health behaviors and health-related outcomes are positive (13).

South Africa's two-tiered health system (23), requires consideration of the different *work environments* and the influence of these settings on the work motivation of clinical pharmacists. However, no significant association was found between the work motivation of clinical pharmacists practicing in either private or public healthcare sector hospitals. Similar to our findings, the type of hospital did not have an influence on the work motivation of medical specialists (14). The hypothesis was that clinical pharmacists employed in the private sector would be more autonomously motivated, as the ratio of pharmacists to patients in the private sector are much higher, and the private sector has created ward-based pharmacist or clinical practice pharmacist posts to accommodate clinical pharmacists. A possible explanation could be that pharmacists who choose to specialize as clinical pharmacists, with the knowledge that no official posts exist, and especially those choosing to work in the public health care sector, with substandard working conditions (51), could already be more intrinsically motivated for the field. This supports the recommendation that more qualitative research is needed to understand the association between the work motivation of clinical pharmacists and their BPN satisfaction.

*Gender* was not related to clinical pharmacists' motivation to practice, which is in contrast with other studies on healthcare professionals which have reported that females are more autonomously motivated than males (45, 47, 52). *Age and work experience* also did not have a significant association with clinical pharmacists' work motivation. This is different from findings of other motivation studies, where specialists younger than 50 years scored higher on AM, and specialists and pharmacists with <15 years' experience scored higher on CM (14, 47). One potential explanation could be that in general the clinical pharmacist respondents' comprise a younger population, the majority with ≤5 years of work experience as compared to the participants in the other studies.

Additional *clinical pharmacy related training* at the current work setting was not related to clinical pharmacists' work motivation. Contrarily, on-site training has previously been found to be a predictor of general motivation in health care workers (53). SDT literature further acknowledges that feeling competent (which additional training can enhance) is crucial for AM. Our findings suggests that the available on-site training may not stimulate specialists' feelings of competence. The offered training options may not be tailored to their needs (47).

Clinical pharmacists who were *not employed in a dedicated specialist position* reported higher amotivation to practice. One reason might be that clinical pharmacists who are not in dedicated positions, cannot optimally fulfill their role in providing adequate pharmaceutical care (27), in turn leading to diminished professional autonomy (being able to determine and set standards for professional practice). This was confirmed by one of the interviewed clinical pharmacists. In line with SDT (12), repeatedly engaging in autonomously motivating activities stimulates AM. Van der Burgt et al. (54) likewise also confirmed that when a specialist has a higher AM for work, it stimulates their daily task-related motivation (in this case, motivation to deliver clinical services).

Another factor significantly associated with the work motivation of clinical pharmacists, was whether they received an *additional income for service delivery*. Findings showed that receiving an income benefit did seem to make a difference, as most clinical pharmacists who were not compensated were found to have higher amotivation for practice. A study on the factors influencing the motivation of clinicians in a low-income country found similar results, where a higher salary was associated with motivation and noted as a prerequisite determinant for any other intervention to change motivation using non-financial ways (55). However, this contradicts the general SDT literature which says that financial incentives harm autonomous motivation in the long run. This is also different from findings showing that external material rewards had the least influence on medical specialists' motivation in the Netherlands (14). We hypothesize that we found this because the clinical pharmacists think that their current income (in non-clinical pharmacist positions) is not commensurate with their qualification. This could have the effect of BPN frustration, especially autonomy frustration. They might be seeing additional income as something they deserve for this imbalance and getting this additional income makes them feel valued. This needs to be tested in further qualitative research.

From the interviews, most participants explained that challenges of not being in a dedicated position include: lack of support from colleagues and management, as well as lack of understanding from other health care professionals regarding their role. Working with colleagues, in a way that is experienced as positive, is motivating as it fulfills that need among clinical pharmacists. On the flipside, working with colleagues can influence motivation negatively when there is diminished feeling of being related to one another (9, 14, 56). In line with these results clinical pharmacists in the present study experienced higher amotivation when they did not feel supported and related to other health care professionals.

### Implications for Practice and Future Research

Our findings demonstrate the existence of CM in clinical pharmacists and shows how the current work environment is driving CM, more than AM. Motivation is a dynamic entity and CM can change into AM and vice versa depending on the degree to which BPNs are being met (13).

In line with literature, this study's findings could provide guidance on how to convert CM to AM by satisfying clinical pharmacists needs for autonomy, competence and relatedness. This in turn could improve the wellbeing among clinical pharmacists, supporting the implementation of clinical services, preventing possible adverse events (burnout and less job turnover intention) and keeping the quality of patient care as high as possible. Avoiding frustration of the BPNs is more important for preventing burnout than ensuring satisfaction of BPNs (10), as moving from autonomous toward controlled motivation is also directly associated with burnout. The thwarting of autonomy and relatedness, by not being appointed in a dedicated clinical pharmacy position, can easily lead to a sense of frustration. The shortage of pharmacists, especially within the COVID-19 pandemic aggravates the situation, as those who do the work are overburdened.

To support clinical pharmacists, measures need to be taken to reinstate their sense of autonomy and relatedness. A supportive work environment where the BPNs are fulfilled will stimulate the most optimal type of motivation for practice. It could furthermore also provide additional positive outcomes, such as enhancing their motivation for lifelong learning. Continuing professional development sequentially helps health care professionals keep up with their changing context, positively affecting the quality of health care and patient safety (14). These implications could also be interesting for other low to middle-income countries where there is a slow uptake of clinical pharmacy services.

Future research on the BPNs among clinical pharmacists is a next step in working toward an environment that stimulates autonomous work motivation. Future qualitative research on relatedness among clinical pharmacists is especially needed to determine how this basic need can be fulfilled. A work context that supports the basic needs satisfaction is perceived to not only stimulate motivation, functioning and wellbeing among health care professionals, but also has benefits for the organization (57). Research shows that specialists experience more autonomy when they organize their own time (14). An environment empowering clinical pharmacists to be autonomous in their time planning, enabling them to devote most of their time to the delivery of pharmaceutical care in the wards and receiving support from colleagues and management could contribute to more AM. Furthermore, an environment with standardized practice guidelines, not only driven by antimicrobial stewardship activities (8), could enable clinical pharmacists to deliver patient care they derive inspiration from.

For sustainable work performance, literature encourages work environments to design compensation systems that avoid measurement and monitoring of performance, but rather fulfill the BPNs, contributing to adaptive and proactive performance and well-being (58). Organizations are encouraged to structure three elements of the work environment: job design, interpersonal relationships/leadership and compensation (59). Autonomy can be encouraged by actively providing clinical pharmacists with opportunities to participate in decision-making. Feelings of relatedness can be encouraged by having positive interactions (on the quality of their relationships) with management and cooperation with colleagues. Competence need satisfaction can be encouraged by providing adequate training.

For future research, it would be interesting to explore the barriers and facilitators/energizers for clinical pharmacists' work motivation and how they cope with the issues that demotivate them. Research on the needs among clinical pharmacists is therefore recommended as a next step in working toward an environment that provides an autonomy, competence, and relatedness supportive work setting for them to develop in and deliver pharmaceutical care services. Based on the study findings, in-depth qualitative research on motivation among clinical pharmacists is necessary, to get more insight into the factors predictive of motivation.

## Limitations

This study used self-report measures, consisting of self-assessment scales where respondents tend to overestimate themselves (60) which could mean results provided are an overestimation of the level of work motivation of clinical pharmacists. Furthermore, the AMS questionnaire has not frequently been used in health care professionals (40, 42), and it has only been validated in high school and college students. Therefore, more experience with this and other scales is necessary to demonstrate the validity of the instrument in practicing health care professionals. Although it was a relative small sample size, considering that the overall population is also small, together with the common knowledge that specialists are sent an overwhelming amount of questionnaires, the response rate was considered good for this population. The study was conducted in the context of a specific country, therefore the findings may not be applicable to other clinical practices. Furthermore, although SDT is a universal theory and has been validated in many life domains, not much is known about the motivation of clinical pharmacists. More research in other healthcare contexts is necessary to determine generalizability of the study findings. Lastly, an additional limitation is the potential effect of the COVID-19 crisis on the results. The second data collection wave took place in 2021, affecting 12% (14; *n* = 114) of participants. The recent pandemic has a known additional strain on health care workers who already perceived their jobs as highly demanding (61).

## Conclusion

Clinical pharmacists had a high mean AM and CM, and low mean amotivation. Higher amotivation was found in graduates who are currently not practicing in dedicated clinical pharmacist posts, as well as higher amotivation in graduates who do not receive additional financial benefits for clinical services. In line with SDT literature, the interviews revealed that relatedness and autonomy are the most important factors for clinical pharmacists' work motivation. A work environment that drives AM, instead of CM can improve professional wellbeing, service implementation and prevent possible adverse events.

## Data Availability Statement

The raw data supporting the conclusions of this article will be made available by the authors, without undue reservation.

## Ethics Statement

The studies involving human participants were reviewed and approved by Sefako Makgatho Health Sciences University Research and Ethics Committee, Sefako Makgatho Health Sciences University. The patients/participants provided their written informed consent to participate in this study.

## Author Contributions

LC contributed to the conceptualization of the study, the intellectual content, analyses, interpretation of data, drafting and revising of the paper, and final approval. AW contributed to the intellectual content in the manuscript, analyses, interpretation of data, revising of the paper, and final approval. EB and AG contributed to the intellectual content in the manuscript, revising of the paper, and final approval. RK contributed to the conceptualization of the study, analyses, interpretation of data, revising of the paper, and final approval. All authors contributed to the article and approved the submitted version.

## Conflict of Interest

The authors declare that the research was conducted in the absence of any commercial or financial relationships that could be construed as a potential conflict of interest.

## Publisher's Note

All claims expressed in this article are solely those of the authors and do not necessarily represent those of their affiliated organizations, or those of the publisher, the editors and the reviewers. Any product that may be evaluated in this article, or claim that may be made by its manufacturer, is not guaranteed or endorsed by the publisher.

## Appendix

**Table A1:** CFA factor loadings on all variables.

**Item**	**Amotivation**	**EM-external regulation**	**EM-introjected**	**EM-identified**	**IM- to experience stimulation**	**IM-toward accomplishment**	**IM- to know**
Q2							0.491
Q9							0.641
Q16							0.721
Q23							0.791
Q6						0.666	
Q13						0.616	
Q20						0.762	
Q27						0.671	
Q4					0.646		
Q11					0.737		
Q18					0.617		
Q25					0.881		
Q3				0.564			
Q10				0.663			
Q17				0.504			
Q24				0.751			
Q7			0.728				
Q14			0.551				
Q21			0.541				
Q28			0.786				
Q1		0.418					
Q8		0.769					
Q15		0.658					
Q22		0.917					
Q5	0.558						
Q12	0.927						
Q19	0.691						
Q26	0.561						

*IM, Intrinsic Motivation; EM, Extrinsic Motivation; Q, Question*.
